# Dynamic Closed-Loop Medical Record Quality Management Using an AI-Driven Multilevel Quality Control System: Development and Implementation Study

**DOI:** 10.2196/80580

**Published:** 2026-07-27

**Authors:** Yuanpeng Wu, Hui Li, Aonan Su, Wanjun Yang, Liping Ding

**Affiliations:** 1 Department of Medical Records and Statistics Zhejiang Provincial People's Hospital/People's Hospital of Hangzhou Medical College Hangzhou China

**Keywords:** medical record quality, AI, 4-level quality control, full-process quality control, closed-loop management

## Abstract

**Background:**

As the core documentation of the clinical diagnosis and treatment process, the quality of medical records is directly related to medical safety and efficiency. However, traditional manual quality control (QC) has limitations such as limited coverage, low efficiency, and inconsistent standards, making it difficult to meet the high standards of modern hospitals.

**Objective:**

The purpose of this study was to develop a medical record QC platform based on AI technology and to build a multilevel QC system relying on the platform to comprehensively improve the form and content of electronic medical records (EMRs).

**Methods:**

This study established a QC rule database based on relevant medical record writing standards, combined deep learning and natural language processing technologies to build an AI-driven QC engine, designed a 4-level QC and management process, and implemented the system in a comprehensive tertiary hospital in China.

**Results:**

After applying AI for the first level of QC, the coverage rate of automated initial screening of EMRs reached 100%. On this basis, combined with manual participation in the second to fourth level of QC, a new model of medical record quality management was formed, featuring human-machine collaboration, interaction between QC units and clinical departments, efficient closed-loop operation, and equal emphasis on form and connotation. The completeness, timeliness, consistency, and accuracy of EMRs significantly improved, while various indicators, such as the grade A rate and excellent rate, continued to increase (all P<.001).

**Conclusions:**

This study demonstrated that the implementation of an AI-based 4-level QC management model helped overcome the limitations of traditional manual QC methods to a certain extent, facilitated comprehensive, dynamic, and closed-loop management of EMRs, and effectively improved the overall quality of medical records. This model can provide a reference for other hospitals seeking to reform their QC models.

## Introduction

With the continuous advancement of medical informatics, electronic medical records (EMRs) have been widely used in medical institutions. As repositories of medical data such as patient disease information, diagnosis processes, and treatment plans, EMRs are not only the cornerstone of clinical decision support systems and medical insurance payments but also the legal basis for handling medical disputes and a source of information for hospital teaching and scientific research. Improving the quality of EMRs plays a key role in ensuring patient safety, enhancing medical quality, and increasing hospital operational efficiency. In recent years, the National Health Commission of China has successively issued a series of relevant documents, including *Key Points of Core Systems for Medical Quality and Safety* [1] and *Quality Control Indicators for Medical Record Management* (*2021 Edition*) [2], which put forward requirements for the quality of medical record writing. The *Action Plan for Comprehensive Improvement of Medical Quality* (*2023-2025*) [3] issued in 2023 lists the medical record quality improvement project as a special action, emphasizing the core tasks of improving the connotation quality, integrity, and timeliness of medical records; strengthening coding management and medical record quality training; and standardizing medical record writing. At the same time, it points out the objectives of improving the quality and safety management system and mechanism while enhancing the degree of refinement and scientificity. Therefore, how to take effective quality control (QC) and management measures to further improve the timeliness, completeness, and accuracy of EMRs has become a focus of attention for many hospitals.

However, the traditional QC of EMRs in China still faces many challenges [4-8]. Multiple platforms, such as the hospital information system (HIS), laboratory information system (LIS), and picture archiving and communication system (PACS), are often independent of each other, and the problem of data islands is serious, which increases the workload of medical record QC. Clinicians’ overreliance on the automation advantages of the EMR system often leads to problems such as input errors and repetitive contextual content, and it is difficult to track and solve such medical record quality risks in real time using a QC model that emphasizes terminal review while ignoring the process. The summary of the various QC outcome indicators mainly relies on manual statistics, which has the disadvantages of being slow speed and poor accuracy, so it is impossible to carry out timely, targeted medical record quality improvement according to comprehensive and accurate feedback. In addition, the weak awareness of clinicians’ standardized writing of medical records, the lack of professional competence of medical record QC staff, and the lack of a related supervision system are also important factors influencing the effect of QC.

To address the aforementioned issues, some studies have explored the optimization of EMR QC methods. Song et al [9] analyzed the internal quality problems of medical records in their hospital by sampling 10 medical records every month and carried out thinking and explored aspects such as medical record writing training, improvement of archiving processes, and team building. Li [10] adopted the Plan-Do-Check-Act cycle method, which includes making plans, strict implementation, repeated inspections, and scientific treatment, for the management of medical record quality problems to improve the situation of medical record defects. A tertiary hospital in Chongqing summarized and processed various indicators and combined data cleaning, quantitative classification, and natural language processing (NLP) technology to achieve automatic detection of medical record QC indicators [11]. Although these measures improved problems with medical record writing to some extent, there were still limitations, such as insufficient use of AI technology, low participation of relevant personnel, and disconnection between the system and management. Therefore, to further enhance the efficiency of QC, this study built an intelligent QC management platform based on technologies such as NLP and machine learning. Relying on the platform, a 4-level QC system and a closed-loop management model were constructed to overcome the limitations of traditional QC and form an innovative AI-assisted QC (AI-QC) paradigm, thereby achieving continuous and systematic improvement in the quality of medical records.

## Methods

### Research Setting and Team Composition

This study was conducted at a large, comprehensive, tertiary-level hospital in Zhejiang Province. The project team consisted of 2 working groups. One was a platform construction group composed of platform architects, information technology experts, and data engineers, which was mainly responsible for the development, maintenance, and optimization of the system. The other was a collaborative management group composed of QC experts, medical record managers, clinicians, and medical information coordinators, which was mainly responsible for medical record quality monitoring, result analysis, and cross-departmental liaison.

### Design and Build an Intelligent Quality Management System

**Figure 1:**
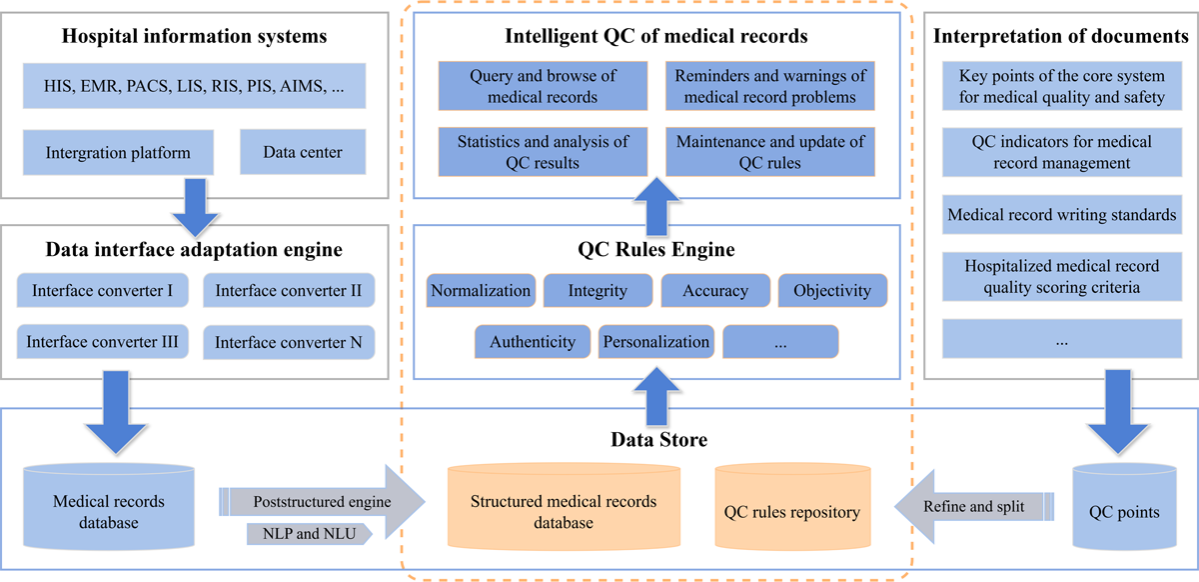
Architecture of an intelligent quality control (QC) management platform. AIMS: anesthesia information management system; EMR: electronic medical record; HIS: hospital information system; LIS: laboratory information system; NLP: natural language processing; NLU: natural language understanding; PACS: picture archiving and communication system; PIS: pathology information system; RIS: radiology information system.

The platform adopted a 3-layer modular design consisting of a data layer, a processing layer, and an application layer, with 3 engines of automated acquisition, intelligent structuring, and rule-based QC as the core (Figure 1). First, the relevant national and provincial regulations and standards for medical record QC were interpreted, and the key QC points were refined and categorized based on the specific needs of our hospital to establish QC rules. A multilayer validation mechanism was used to ensure the rationality and feasibility of these rules: the collaborative management group conducted multiple rounds of professional reviews to confirm clinical applicability and standardization; the platform construction group performed retrospective testing on historical medical records to verify technical executability and logical accuracy; and pilot operations were carried out in selected departments to evaluate the practical application of the rules, with dynamic adjustments made to the rule points based on feedback from clinicians. As a result, a customizable and optimized QC rule repository was formed, covering dimensions such as timeliness, completeness, consistency, standardization, and objectivity, with each rule assigned a specific deduction score. Second, the medical record database was formed by integrating data from existing hospital systems, such as EMR, HIS, LIS, PACS, and the surgical anesthesia system. Poststructured processing of medical record documents, such as free text, pathology reports, and test results, was performed using NLP techniques such as medical word segmentation, entity recognition, multimodal data alignment, and contextual semantic understanding [12,13]. The entity types and attributes were defined based on international and domestic clinical standard terminology, while cross-modal knowledge extraction and relationship establishment were combined with deep learning methods to form a disease knowledge graph [14,15]. Finally, using the QC rules engine, real-time analysis, comparison, judgment, and feedback for noncompliant or discrepant medical record content were carried out, and a visualization module for QC results was developed to support auxiliary clinical applications, QC management, and medical research.

Meanwhile, during the system construction process, we carefully set the minimum score threshold for medical record submission by comprehensively considering national industry norms, policy requirements, and the risk management needs of our hospital. The specific basis is as follows. First, the *Basic Norms for Medical Record Writing* issued by the National Health Commission of China in 2010 require that medical records meet core quality criteria, such as completeness, timeliness, and standardization [16]. On the basis of these guidelines, medical institutions have developed quantitative scoring tables, typically setting 90 points as the passing threshold for grade A medical records, indicating that the records contain no major defects [17,18]. This score has become a widely accepted standard in China. Second, the *Evaluation Standards for Tertiary Hospitals* (*2022 Edition*) explicitly stipulate that medical institutions must undergo regular quality assessments, with the compliance rate for grade A medical records serving as a core evaluation indicator [19]. To actively respond to the national evaluation policy, our hospital needed to set corresponding score requirements for medical record submission. Finally, our hospital’s internal data reports over the years indicated that medical records with scores below 90 points were significantly associated with higher coding error rates, insurance claim rejection risks, and medical dispute occurrence rates. To strengthen risk prevention and control, this study raised the medical record submission threshold from 90 points to 92 points to further improve the overall quality of medical record writing. Additionally, it should be noted that current AI technology cannot fully replace human judgment in identifying all potential issues. Therefore, even if a record passes the AI-established threshold of 92 points, it is still possible for it to be ultimately classified as a grade B record following manual review.

### Form a Medical Record QC Process

Through the operation of the rule base in the intelligent QC system for medical records, the full-process QC of EMRs was achieved, including reminders before writing, supervision during the process, and review after completion. When clinicians start writing medical records, they are intelligently reminded of the QC rules applicable to the documents being created, and time-sensitive management is implemented for instruments with time-limited requirements. In the process of writing medical records, real-time monitoring of the writing situation is carried out according to the preset QC rules, and early warnings and prompts are provided for the identified problems to achieve continuous supervision, reminders, and feedback to improve the standardization of writing. At the end of writing, a cutoff value of 92 points is used to assess whether the record can be submitted, thus reducing the number of single-item rejected medical records. After the medical records are submitted, based on intelligent QC rules and assisted by manual screening, automatic feedback and manual revision are carried out for problematic medical records to complete the final QC.

### Establish a 4-Level QC System and Closed-Loop Management Model

**Figure 2:**
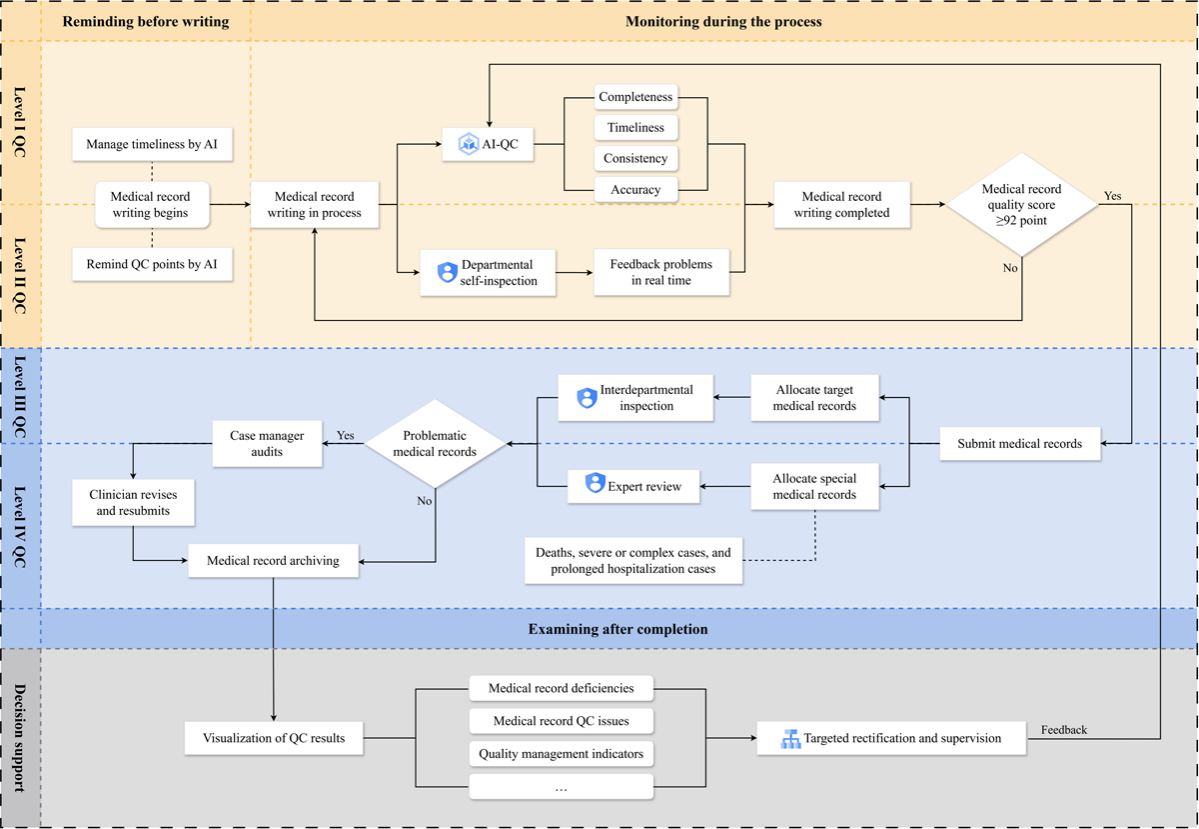
The 4-level quality control (QC) system and management model of AI-driven electronic medical records (EMRs). AI-QC: AI-assisted quality control; level I QC: the first level of quality control; level II QC: the second level of quality control; level III QC: the third level of quality control; level IV QC: the fourth level of quality control.

The QC management model was optimized based on the intelligent QC management platform and the full-process QC method of EMRs (Figure 2).

The first level of QC was AI review, which embedded the rule engine into the physician’s medical record writing system and intelligently verified various medical record documents, such as admission records, checkup records, surgical records, and informed consent forms. When missing key fields, conflicting time nodes, and nonstandardized terminology were detected, reminders and warnings were provided through hovering pop-up windows to avoid the generation of grade C medical records. The second level of QC was departmental self-inspection, which relied on the QC management platform to formulate a sampling allocation strategy for medical records in operation and conducted in-depth intradepartmental self-inspection of medical record documents with the medical team leader as the core. After writing problems were identified, the results of the inspection were fed back to the physicians in real time through the platform, and they were encouraged to make timely corrections, thereby effectively reducing the incidence of grade B medical records. The third level of QC was an interdepartmental inspection. Through the QC management platform, a random sampling strategy for discharge medical records was generated based on predefined department matching rules, enabling cross-departmental audits. These matching rules were collaboratively established by medical record QC specialists, medical record management personnel, and clinical department directors (Multimedia Appendix 1). After the results of mutual checking were fed back to the case management team, the case managers carefully reviewed the records and returned the medical records with problems to the clinical team for correction, thereby improving the excellent rate of medical record quality. The fourth level of QC consisted of expert review, and the experts from the medical record management committee carried out spot checks of the discharge medical records of patients who had died, had severe and complex conditions, or were hospitalized for a long time. At this stage, QC mainly focused on the internal quality of EMRs, including the accuracy of diagnosis, comprehensiveness of condition analysis, and rationality of treatment plans, drawing on the rich theoretical knowledge and clinical experience of experts to further strengthen the internal quality of medical records.

At the end of the monthly 4-level QC work, the case manager used the dashboard in the QC management platform to conduct intelligent statistical analyses of the medical record defect details, document quality scores, and other indicators, and visualize them according to different departments, medical groups, and QC nodes. They then took corresponding corrective measures depending on the results of the analysis, including perfecting the QC points, clarifying the reward and punishment mechanism, and updating the relevant assessment system. At the same time, the implementation effects of these management decisions were recorded and analyzed by the system, which guided the focus of QC in the next cycle. Eventually, a closed-loop scientific management model was formed, covering problem monitoring, intelligent analysis, precise intervention, and effect tracking.

### Statistical Analyses

In this study, categorical data were expressed as frequencies and percentages. The Cochran-Armitage trend test was used to analyze changes in medical record quality indicators over time. To further evaluate the practical clinical and managerial significance of the improvements, the absolute risk difference and its 95% CI were also calculated, with the 95% CI estimated using the Newcombe method. All analyses were performed using R software (version 4.2.1; R Foundation for Statistical Computing), and a 2-sided test of *P*<.05 was considered statistically significant.

### Ethical Considerations

This study was approved by the Ethics Committee of Zhejiang Provincial People’s Hospital (QT2025135), which waived the requirement for written informed consent because the procedures were part of routine care and did not involve personal or private information. No financial compensation was provided, as this study did not involve direct participant enrollment.

## Results

**Figure 3:**
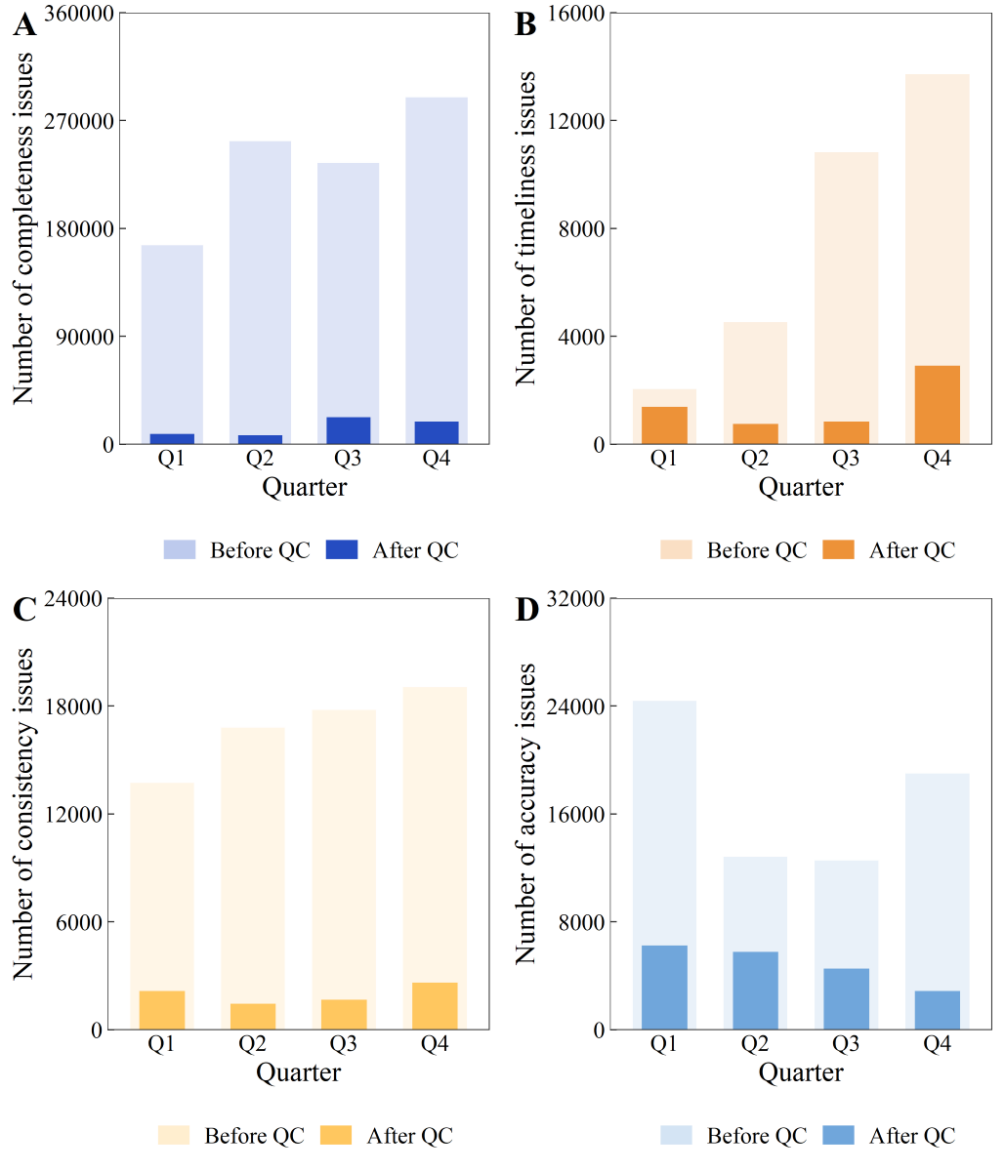
Improvement in various types of problems in electronic medical records (EMRs) after AI-assisted quality control (QC) in 2024. (A) completeness, (B) timeliness, (C) consistency, and (D) accuracy. Q1: first quarter (January-March); Q2: second quarter (April-June); Q3: third quarter (July-September); Q4: fourth quarter (October-December).

Since its application, the 4-level QC management model for EMRs based on AI has gradually covered all clinical departments of the hospital. It formulated 285 standardized QC rules and 2393 related QC points, achieving 100% QC coverage of the documents. The 4 major issues of completeness, timeliness, consistency, and accuracy in EMR writing significantly improved after AI-QC (Figure 3). Several indicators also significantly improved. For instance, compared with December 2023, the 2-day archiving rate of discharged patients in December 2024 reached 98.10%, an increase of 2.86% year over year. Meanwhile, the signing rate of informed consent forms rose from 99.55% to 99.75%, and the incidence of unreasonable medical record copying dropped from 3.26% to 0.69%. The quality assessment results of the randomly inspected special medical records showed that, with the passage of time across the 3 stages before, during, and after the application of the 4-level QC system, the rate of grade A (*z* score=13.64; *P*<.001) and the excellence rate (*z* score=3.88; *P*<.001) of medical record quality showed an upward trend. The rate of grade B (*z* score=−13.64; *P*<.001) showed a significant downward trend (Table 1). The effect size analysis (Table 2) indicated that the proportion of grade A increased by 9.34% (95% CI 5.34%-13.28%) in the early stage and further increased by 15.61% (95% CI 12.93%-18.46%) in the later stage, resulting in a cumulative improvement of 24.95% (95% CI 21.92%-28.06%). Correspondingly, the proportion of grade B cumulatively decreased by 24.95% (95% CI −28.06% to −21.92%). The excellent rate improved by 2.40% (95% CI 0.56%-5.40%) in the early stage and continued to rise by 3.96% (95% CI 0.50%-7.45%) in the later stage, achieving a cumulative increase of 6.36% (95% CI 3.13%-9.67%).

**Table 1:** Comparison of quality indicators for special medical records before and after implementation of the 4-level quality control system.

Indicator	Before (July-December 2023; n=841), n (%)	After (January-June 2024; n=760), n (%)	After (July-December 2024; n=720), n (%)	*z* score	*P* value
Grade A rate	623 (74.08)	634 (83.42)	713 (99.03)	13.64	<.001
Grade B rate	218 (25.92)	126 (16.58)	7 (0.97)	−13.64	<.001
Excellent rate	75 (8.92)	86 (11.32)	110 (15.28)	3.88	<.001

**Table 2:** Effect size analysis of improvements in medical record quality.

Indicator	Early improvement (January-June 2024 vs July-December 2023), ARD^a^ (%; 95% CI)	Sustained improvement (July-December 2024 vs January-June 2024), ARD (%; 95% CI)	Cumulative improvement (July-December 2024 vs July-December 2023), ARD (%; 95% CI)
Grade A rate	9.34 (5.34 to 13.28)	15.61 (12.93 to 18.46)	24.95 (21.92 to 28.06)
Grade B rate	−9.34 (−13.28 to −5.34)	−15.61 (−18.46 to −12.93)	−24.95 (−28.06 to −21.92)
Excellent rate	2.40 (0.56 to 5.40)	3.96 (0.50 to 7.45)	6.36 (3.13 to 9.67)

^a^ARD: absolute risk difference.

The hospital incorporated training on medical record quality into regular management, including regularly holding specialized training lectures, conducting discussions and learning sessions on medical record quality scoring norms, and organizing medical record knowledge competitions. It also developed the Medical Record Quality and Management Manual and released it on the enterprise WeChat (Tencent Holdings Limited) and the hospital’s online platform. In addition, the hospital clearly defined multiple requirements for medical record writing, reward and punishment schemes, and supervision and feedback systems, and improved the corresponding standard documents, such as the Medical Record Quality Management System and the Medical Record Quality Assessment System.

## Discussion

The quality of medical records is one of the key indicators for performance assessment of national public hospitals and an important reflection of the medical level and comprehensive strength; ensuring and improving the quality of medical records has always been a core task of hospitals at all levels. To overcome the shortcomings of the previous QC model, we established and implemented a 4-level QC and management system based on AI in a tertiary public hospital in Zhejiang Province.

The results of this study showed that after the implementation of the AI-assisted 4-level QC management model, the completeness, timeliness, consistency, and accuracy of EMR writing were effectively improved, and multiple QC indicators of medical records increased, with the overall quality of medical records steadily improving. The effect size analysis further quantitatively supported the practical value of the model at the clinical and management levels. The rate of grade A medical records cumulatively increased by 24.95%, indicating that the basic quality of medical documents was systematically enhanced, which was directly related to the reduction of medical safety and compliance risks. At the same time, the excellent rate cumulatively increased by 6.36%, indicating that quality improvement surpassed the defect correction stage and was steadily moving toward deeper internal quality and excellence standards. These achievements benefited from the in-depth application of information technology and the collaborative optimization of the management system. First, the intelligent QC platform was able to conduct a census of the entire hospital’s operating medical records, with 100% QC coverage of EMRs. The preset QC rules engine in the system provided real-time monitoring, reminders, and feedback for medical record problems. This system extended QC from retrospective to concurrent and prospective stages, improving the previous situation of focusing on the end rather than the process. It comprehensively and efficiently reviewed problems that existed in the entire process of medical records from creation to submission. Examples include first attending physician checkup records that were not completed within 48 hours; missing pathology report results in the course of the records; entering the “left side” in the disease information incorrectly as “right side”; and discharge times on the first page that were inconsistent with those in the discharge record [20]. Second, compared with the single-level model in previous studies, which only combined AI-QC with random checking by case managers, the multilevel QC process we constructed had unique advantages. The millisecond response efficiency and session intervention of the first level of QC not only controlled the quality of medical records from the source but also significantly reduced human resource requirements and improved the efficiency of QC. The second level of QC further identified the rationality of the diagnosis and treatment logic that was difficult for AI to determine through the self-inspection mechanism of the medical team. At the same time, it highlighted the supervisory responsibility of the medical team leader and encouraged physicians within the team to actively participate in medical record management. In the third level of QC, surgeons were more likely to identify logical loopholes in documentation, such as the assessment of surgical indications and prevention of postoperative complications, when checking the medical records of physicians, whereas physicians paid more attention to the rationality of perioperative medication and the continuity of chronic disease management when reviewing surgical records. Such complementary review perspectives resulting from professional differences helped overcome the limitations of self-checking and expanded both the breadth and depth of medical record QC. At the fourth level of QC, the in-depth analysis of special medical records by experts accurately identified detailed and underlying errors, such as insufficient diagnostic basis, missing differential diagnoses, and diagnostic and treatment plans that were not in line with the latest guidelines, thereby promoting further enhancement of the internal quality of EMRs. Finally, under the closed-loop scientific management model, we implemented targeted rectifications for key areas, such as standardizing physicians’ medical record writing, building a medical record management team, and improving standardized systems, which complemented the QC process and ultimately contributed to the continuous improvement of quality and the dynamic optimization of management.

We accumulated extensive operational experience in the practical application of the 4-level QC management model and developed corresponding plans for ongoing maintenance and optimization of the system. At present, this project plans to deploy DeepSeek, Qwen (Alibaba Cloud), and other large language models (LLMs) in the intelligent QC management system (Multimedia Appendix 2) to further enhance the system’s ability to analyze complex semantics, cross-paragraph logical contradictions, and unstructured information in medical records. At the technical implementation level, the key steps include building a multimodal interface via middleware to enable data integration with the HISs, performing data cleaning on historical medical records and constructing an annotated corpus, fine-tuning the model by incorporating QC rules and clinical requirements, and conducting regular maintenance and optimization through expert feedback and incremental training. In terms of functional advantages, compared with traditional language tools, LLMs can more accurately parse complex medical terms and ambiguous expressions and leverage long-distance context reasoning to discover hidden defects such as disconnection between medical orders and examination results or conflicts in drug interactions, which are difficult to detect [21,22]. Their incremental learning and dynamic fine-tuning mechanisms enable the model to continuously adapt to updated diagnostic and treatment guidelines and clinical needs [23,24]. In recent years, LLMs such as ChatGPT (OpenAI), Qwen, and DeepSeek have demonstrated outstanding performance in disease diagnosis and diagnostic reasoning [25-27], providing valuable theoretical references for our hospital’s deployment of LLMs. At the same time, relevant documents issued in China on data security and ethical review provided policy guarantees for the implementation of this plan within the compliance framework [28-30]. We believe that continued optimization of the system performance will provide more efficient and accurate intelligent solutions for the quality management of EMRs in the future and elevate the quality of medical care to a new height.

This study has some limitations. First, as a systematic reform implemented in a real medical setting, this study adopted a single-group pre-post design without a parallel control group. During the study period, in addition to introducing the AI-QC system, the hospital simultaneously implemented multiple synergistic interventions, such as training programs and incentive mechanisms, and formed a corresponding management model. At the same time, confounding factors such as updates to the HIS and improvements in user proficiency may have been present during the observation period. Therefore, this study was unable to distinguish the contribution of the AI-QC system from the effects of concurrent measures and other confounding factors and could not draw conclusions regarding causal effects. Second, although the QC management model implemented at Zhejiang Provincial People’s Hospital was effective, the results were limited to a single hospital, and the generalizability of the findings requires further validation. We believe that the concept of a multilevel QC framework and the closed**-**loop management model presented in this study have strong cross**-**institutional applicability and can be promoted as a general management framework, while the deployment of the AI platform is highly dependent on a hospital’s IT infrastructure. Therefore, in practical implementation, the heterogeneity of different health care settings must be fully considered, with targeted adjustments made accordingly. For primary community hospitals with weak digital infrastructure, the QC system would need to be adjusted toward a lightweight and modular design, support low-cost and cloud-edge collaborative computing, and focus on basic needs such as chronic disease management and public health services. For health care institutions in the early stages of informatization, a phased implementation path should be designed to progressively enhance multisource heterogeneous data integration capabilities and nonstandardized data processing. For tertiary hospitals with mature information systems, emphasis should be placed on deep integration with existing complex hospital systems and the fine-grained configuration of specialized QC rules. In international applications, regionalized configurations must be developed based on differences in medical standards, relevant regulations, and disease profiles. In the future, we need to collaborate with more medical institutions, through multicenter and multilevel verification to further optimize the elasticity and scalability of this model. Finally, with the rapid development of individualized and specialized diagnosis and treatment approaches at the present stage, hospitals’ attention to the QC of medical records for specialized diseases has gradually increased [31,32]; however, our QC system is slightly insufficient in this aspect of in-depth vertical development. In the future, under the premise of meeting the application requirements of medical record QC, our study will formulate personalized connotative QC rules based on different disciplines, different diseases, and different diagnosis and treatment methods for individual diseases, further optimize the QC system, and extend the management model toward greater refinement.

In conclusion, this study constructed a new model of closed-loop QC management with AI technology as the foundation and 4 levels of QC as the structure, which facilitated the comprehensive enhancement of the quality of medical records and the continuous improvement of case management, and can provide a reference for other hospitals to promote the reform of the QC system. At the same time, the QC of medical records is a complex and long-term task, and it is still necessary to observe and summarize the shortcomings of the model in practical applications, make timely corrections and improvements, and continuously explore a better QC management solution for EMRs.

## Data Availability

The datasets used and analyzed during the current study are available from the corresponding author (LD) on reasonable request.

## Appendix

Multimedia Appendix 1 Departmental mutual inspection matching list.

Multimedia Appendix 2 Deployment plan for a large language model.

## Abbreviations

AI-QC: AI-assisted quality control
EMR: electronic medical record
HIS: hospital information system
LIS: laboratory information system
LLM: large language model
NLP: natural language processing
PACS: picture archiving and communication system
QC: quality control
