# Supplementary material for: Dynamic Closed-Loop Medical Record Quality Management Using an AI-Driven Multilevel Quality Control System: Development and Implementation Study
Source: JMIR Med Inform. 2026 Jul 27;14:e80580. doi: 10.2196/80580 (PMC13458350; doi:10.2196/80580)
Supplement: Supplementary file 1 [file medinform_v14i1e80580_app1.docx]

| **No.** | **Department** | **Paired Department** |
| --- | --- | --- |
| 1 | Department of Obstetrics | Department of Gynecology |
| 2 | Department of Gynecology | Department of Obstetrics |
| 3 | Department of Pediatrics | Department of Medical Oncology |
| 4 | Department of Medical Oncology | Department of Pediatrics |
| 5 | Department of Otorhinolaryngology | Department of Head and Neck Surgery |
| 6 | Department of Head and Neck Surgery | Department of Otorhinolaryngology |
| 7 | Department of Radiation Oncology | Department of Nuclear Medicine |
| 8 | Department of Nuclear Medicine | Department of Radiation Oncology |
| 9 | Department of Rheumatology and Immunology | Department of Endocrinology |
| 10 | Department of Endocrinology | Department of Rheumatology and Immunology |
| 11 | Department of Hepatobiliary and Pancreatic Surgery and Minimally Invasive Surgery | Department of Gastrointestinal and Pancreatic Surgery |
| 12 | Department of Gastrointestinal and Pancreatic Surgery | Department of Hepatobiliary and Pancreatic Surgery and Minimally Invasive Surgery |
| 13 | Department of Infectious Diseases | Department of Geriatric Medicine |
| 14 | Department of Geriatric Medicine | Department of Infectious Diseases |
| 15 | Department of Emergency Medicine | Department of Critical Care Medicine |
| 16 | Department of Critical Care Medicine | Department of Emergency Medicine |
| 17 | Department of Orthopedics | Department of Hand Surgery and Reconstructive Surgery |
| 18 | Department of Hand Surgery and Reconstructive Surgery | Department of Orthopedics |
| 19 | Department of Colorectal Surgery | Department of Urology |
| 20 | Department of Urology | Department of Colorectal Surgery |
| 21 | Department of Respiratory Medicine | Department of Cardiology |
| 22 | Department of Cardiology | Department of Cardiovascular Surgery |
| 23 | Department of Cardiovascular Surgery | Department of Respiratory Medicine |
| 24 | Department of Interventional Medicine | Department of Plastic Surgery |
| 25 | Department of Plastic Surgery | Department of Interventional Medicine |
| 26 | Department of Rehabilitation Medicine - Critical Care Rehabilitation Unit | Department of Nephrology |
| 27 | Department of Nephrology | Department of Rehabilitation Medicine - Critical Care Rehabilitation Unit |
| 28 | Department of Dermatology | Department of Traditional Chinese Medicine |
| 29 | Department of Traditional Chinese Medicine | Department of Dermatology |
| 30 | Department of General Practice | Department of Pain Management |
| 31 | Department of Pain Management | Department of General Practice |
| 32 | Department of Breast Surgery | Department of Reproductive Endocrinology |
| 33 | Department of Lymphatic Microsurgery | Department of Breast Surgery |
| 34 | Department of Reproductive Endocrinology | Department of Lymphatic Microsurgery |

**Multimedia Appendix 1.** Departmental mutual inspection matching list

| **No.** | **Department** | **Paired Department** |
| --- | --- | --- |
| 35 | Department of Hernia Surgery | Department of Vascular Surgery |
| 36 | Department of Vascular Surgery | Department of Hernia Surgery |
| 37 | Department of Neurology | Department of Neurosurgery |
| 38 | Department of Neurosurgery | Department of Neurology |
| 39 | Department of Gastroenterology | Department of Hematology |
| 40 | Department of Hematology | Department of Gastroenterology |
| 41 | Department of Thoracic Surgery | Department of Ophthalmology |
| 42 | Department of Ophthalmology | Department of Thoracic Surgery |
